# Is ambiguity tolerance malleable? Experimental evidence with potential implications for future research

**DOI:** 10.3389/fpsyg.2015.00619

**Published:** 2015-05-18

**Authors:** Megan L. Endres, Richaurd Camp, Morgan Milner

**Affiliations:** Department of Management, Eastern Michigan UniversityYpsilanti, MI, USA

**Keywords:** ambiguity tolerance, experiment, personality, self-efficacy, structured interview

## Abstract

We conducted two research studies to address the malleability of tolerance of ambiguity (TA) by manipulating situational ambiguity. Students participated in a semester-end assessment of their management skills (*n* = 306). In Study 1, students in low and moderate ambiguity conditions had significantly higher post-experiment TA, more positive change in self-efficacy, and marginally higher faculty ratings. In Study 2, a control group (*n* = 103) did not participate in the assessment and was established for comparison to the first study results. The Study 2 students reported TA significantly lower than Study 1 students in the low and moderate ambiguity conditions. The control group TA was not significantly different from that of the Study 1 high ambiguity condition. This further suggested TA’s situational malleability, as those who had controlled access to structured information appeared to have increased their TA over that observed in the other two groups. These results suggest that TA may be malleable. We review the relevant literature, offer hypotheses, report our analyses and findings, and then propose future research, and potential prescriptive applications in such areas as management development, assessment, and decision-making.

## Introduction

Tolerance of ambiguity (TA) is defined as the preference for ambiguous situations (Frenkel-Brunswick, 1948, 1949, 1951). After decades of research, TA continues to be upheld as a critical variable in applied research as “increasingly necessary in the global workplace as socioeconomic forces stretch managers’ capacities to perceive, interpret, and act on environmental information due to rapid globalization, technological advancement, and workforce diversity” (Herman et al., 2010, p. 54).

Despite a long history of research attention to TA and its application, its validity is still in question. For example, TA was first introduced as an attitude a person holds toward objects or situations (Frenkel-Brunswick, 1948, 1949, 1951), but it has primarily been studied as a single factor stable trait (Furnham and Marks, 2013). TA research is characterized by an overall lack of construct development (Herman et al., 2010; Furnham and Marks, 2013) and weakness of its operationalizations (Grenier et al., 2005; McLain, 2009). In fact, Furnham and Ribchester (1995, p. 179) summarized the TA literature as “scattered and diffuse.”

The TA literature is also plagued with an overwhelming preference for correlational studies and a lack of experimental design (Furnham and Marks, 2013), a problem personality research encounters in general (Huang and Ryan, 2011). The lack of TA validity across studies has led to recent questions whether it is truly a stable personality trait or an attitude that varies by context (e.g., Durrheim, 1998).

The purpose of the present study was to investigate the stability of TA in an experimental manipulation of task ambiguity. We conducted two studies in order to examine the malleability of TA shown by senior business students in a capstone-course simulation and the impact of varied task ambiguity on TA. We present hypotheses, method, measures, and results. Last, we discuss implications for research and practice.

## Literature Review

In order for a construct to be considered a stable trait, compelling research evidence is needed across contexts, or as Mischel (2004, p. 2) stated, “finding the invariance in variability.” In the case of TA, the tolerance trait should be stable across tasks and situations with varied ambiguity. An ambiguous context is defined as one in which participants lack information they need to understand the situation or possible outcomes (Furnham and Marks, 2013, p. 718). Frenkel-Brunswick (1949) first identified TA as an attitudinal construct, describing it as varied within the person depending on the focus of the tolerance.

Although original measures of TA were unidimensional, more recent measures have included multiple contexts in which ambiguity may exist, such as in family or work situations (e.g., McLain, 1993, 2009; Furnham, 1994; Durrheim and Foster, 1997; Herman et al., 2010). This modern contextual approach has often focused on individual reactions to ambiguity in varied situations or tasks (e.g., Judge et al., 1999; Endres et al., 2009; Weisbrod, 2009; Herman et al., 2010). However, the contextual approach to studying TA most often still assumes within-person trait stability.

Furthermore, measures of TA have tended to be psychometrically weak and have varied widely in their underlying dimensions (Herman et al., 2010; Furnham and Marks, 2013). However, a few researchers have hypothesized and found within-person variation that suggests TA may be context-specific rather than trait-based (e.g., Glover et al., 1978; Durrheim and Foster, 1997; Durrheim, 1998).

First, Glover et al. (1978) studied TA levels in students before and after a cross-cultural simulation wherein the students had to deal with a large amount of ambiguity. A control group did not participate in the simulation. Prior to the simulation, experimental, and control groups’ TA did not differ. Post-simulation TA levels of the experimental group were significantly higher than the post-simulation TA levels of the control group.

In one of the few experimental studies hypothesizing changes in TA, Durrheim and Foster (1997) found within-person variability across different content areas. These content areas, such as political authorities and family, were specified in the authors’ Attitudinal Ambiguity Tolerance scale (AAT). In a similar study, Durrheim (1998) also found variability in the relationship of TA and conservatism across content domains.

In longitudinal studies, some researchers found that TA changed over time. Studies of age and TA have indicated some change in the construct over time and with experience. Helson and Wink (1992) surveyed 118 working women at age 45 and then at age 52 and found a significant increase in participants’ TA over the 7-years period. Similarly, Howard and Bray (1988) found that managers’ TA increased significantly over time.

Other researchers suggested within-person changes in TA without evidence. For example, researchers have repeatedly stated that international experiences increase TA (e.g., Lashbrooke et al., 2002), or that intercultural training increases TA (e.g., Harris, 1979). There is no experimental evidence to support these claims, however, (Tucker et al., 2011).

In short, TA is a prolific topic of research studies with a variety of contexts and playing multiple roles. Overwhelmingly, the construct has been assumed to be a stable trait, most often studied at one point in time in correlational research (Furnham and Marks, 2013). Only a few experimental manipulations exist to test within-person variability in TA. We do not offer specific hypotheses, but a research goal to further investigate the within-person variability in TA.

## Study 1

The purpose of Study 1 was to design an experiment in which subjects would face varied levels of ambiguity. We expected controlling ambiguity in an experimental manipulation would provide valuable information about whether TA would significantly differ in each group. In addition, we measured pre-experiment self-efficacy and post-experiment self-efficacy due to strong evidence supporting that TA and self-efficacy are positively related (Endres et al., 2009).

### Sample

A total of 324 subjects (55.0% male) participated in an experiment^1^. The average subject age was 23.91 (SD 4.31). Students were from eight class sections of the senior-level capstone management class, and the experiment was implemented in the half-day assessment required of all management majors. Most (69.4%) students were management majors, while the remaining were either management minors (18%) or other business majors (12.6%) taking the course as an elective.

### Methods and Measures

The assessment took place over a 3-h period and included two parts: a management skills interview and a group case analysis. The faculty assessment director visited the student participants’ classes one month before assessment to explain specifics of participation and to hand out a Harvard case study. Students were told to read and take notes on the case, to dress in business casual clothing and to arrive at a specific time.

Student participants were randomly assigned to 35 teams, with four to five students on each team. We randomly assigned teams to one of three conditions of low, moderate and high ambiguity: (1) structured interview of past experiences, or low ambiguity (*n* = 127), (2) structured interview of hypothesized future experience, or moderate ambiguity (*n* = 94), or (3) unstructured interview, or high ambiguity (*n* = 103).

Management literature supports that the strengths of structured interviewing techniques are their lessening of ambiguity about requirements (Campion et al., 1997). Structured techniques (as opposed to unstructured) provide clearer instructions, more information, and follow a format that interviewees expect (Williamson et al., 1997). Structured interviews should lessen ambiguity about the process and allow more focus on the task. Unstructured interviews provide higher ambiguity to subjects than structured interviews. Unstructured interviews’ lack of a common format and identifiable goals make individuals unsure of the purpose of the interview, even as they feel high levels of ambiguity and focus on the process, rather than the task (e.g., Dibner, 1958).

We also randomly assigned faculty evaluators to each of the three experimental conditions and, in some cases, a graduate student assisted in evaluating. Before the assessment, we trained both faculty and graduate students regarding the case study and procedures. We used the evaluations prepared by faculty in our analyses as the measure of performance, although we used the graduate students’ evaluations to establish inter-rater reliability.

On assessment day, students were directed to their groups in separate classrooms where they first filled out pre-assessment questionnaires. Next, students filled out a written interview answer sheet to prepare them for their subsequent oral interview. For the *unstructured interview* group (high ambiguity), the answer sheet questions included:

(1) Outside of today’s experiences, what are your key management skills that you would like us to know about?
(2) How do you demonstrate to someone else that you have these skills?

For the *structured past interview group* (low ambiguity), the answer sheet asked:

(1) Describe a situation in which you applied a management skill in the past.
(2) Describe the action you took (2–3 sentences).
(3) Describe the result of your action (2–3 sentences). What was the outcome? How did you measure it?

For the *structured future interview group* (moderate ambiguity) the answer sheet asked:

(1) Describe a situation in which you may apply management skills in the future.
(2) Describe the action you would take (2–3 sentences).
(3) Describe the likely result of your action (2–3 sentences). How would you measure the outcome?

The students had 10 min to finish the answer sheets after which the faculty evaluator asked questions of each student regarding his/her answer sheet. We trained the faculty to focus the discussion on skill-based feedback and to limit the scope to the specific questions asked of the students on the worksheets. Students then discussed the Harvard case as a team with the goal of delivering a written solution in 50 min. As the last step in the exercise, the faculty member debriefed the team’s work, and the students then filled out the post-assessment questionnaire.

#### Change in Self-Efficacy

We computed “change in self-efficacy” as the difference between pre- and post-experiment self-efficacy. While we used the same questions on the pre- and post-versions of the measure, they were phrased either in the present (“I am confident that I can…today”) or future (“I am confident that I can…in future situations”). Students answered using a scale of 0–100% certainty in the statement. Reliability for pre-experiment self-efficacy was high (α = 0.86), as was reliability for post-experiment self-efficacy (α = 0.90). **Table 1** includes each measure, the scale, number of items, an example question, and reliability (Cronbach, 1951).

**Table 1:** Measures and reliability.

Variable	Scale	Example question	Reliability (α)	# of Items
Pre-task self-efficacy	0–100	In assessment today, I am confident that I will be able to manage conflict effectively.	0.86	12
Post-task self-efficacy	0–100	In future situations similar to assessment, I am confident that I can manage conflict effectively.	0.90	12
Perceived value of assessment process	1–5 (strongly disagree to strongly agree)	I have a better understanding of how to apply the skills I learned as a Management major when I take a professional job as a result of participating in assessment.	0.81	5
Rating of student performance^a^	1–5 (poor to excellent)	Rated in five skill areas: (1) Problem solving/critical thinking (2) Interpersonal/team skills (3) Change management skills (4) Communication skills, and (5) Leadership skills.	0.93	5
Tolerance of ambiguity (TA)	1–7 (strongly disagree to strongly agree)	I prefer familiar situations to new ones (Reversed).	0.83	6

^a^The same rating form was used for the faculty rating, self-rating, and peer rating.

#### Performance

Faculty rated the students’ performance in the overall assessment. The assessment included performance ratings on a five-point scale (1 = “poor,” 5 = “excellent”; of five management skill areas that were consistent with the learning goals set for graduates in this major: (1) problem solving/critical thinking; (2) interpersonal/team skills; (3) change management skills; (4) communication skills; and (5) leadership skills. Reliability was high (0.93) and justified averaging the five questions. Students used the same form to rate their own performance as well.

For 128 of the 324 total subjects, a trained graduate student independently rated student performance using the evaluation forms the faculty evaluators also used. We used these 128 subject ratings to calculate inter-rater reliability with an intraclass correlation, or ICC (McGraw and Wong, 1996), which is considered to be acceptable at 0.79. This ICC helped validate the performance measure, despite there being a single evaluator.

#### Tolerance of Ambiguity

In the post-assessment questionnaire, TA was measured using six items from Budner’s (1962) measure, and a Likert-type scale from 1 (*strongly disagree*) to 7 (*strongly agree*). The scale displayed good reliability (0.83) and a one-factor solution using principle component (PCA) analysis (eigenvalue = 3.24; 53.93% of variance).

#### Opinion of Assessment

In the post-assessment questionnaire, students rated perceived value of the assessment to their careers in five questions on a Likert-type scale ranging from 1 (*strongly disagree*) to 5 (*strongly agree*). The scale displayed good reliability (0.81).

We gathered demographics as potential covariates (gender, age, and years of work experience), number of group mebers, and numbered and coded teams. None of these control variables was significantly related to the outcome measures.

### Results

**Table 2** shows the correlations and descriptives of the main study variables. The sample size varied due to participation that may have varied due to lateness or lost forms. **Table 2** shows that the average TA in the sample was slightly above the scale mean, at 4.03 (SD = 1.24). The average self-efficacy change was 4.05% (SD = 5.40). Faculty ratings, self-ratings, and student post-assessment opinions were close to the maximum five rating and lack of variation may limit the ability to find group differences. Due to the categorical nature of the experimental manipulation variable, more statistical testing must be done to conclude group differences.

**Table 2:** Descriptives and correlations.

	Mean	SD	*n*	1	2	3	4	5
(1) Experimental group	1.98	0.78	326	–				
(2) TA	2.94	1.24	315	0.14^∗^	–			
(3) Self-efficacy	4.05	5.40	312	0.12^∗^	-0.02	–		
(4) Faculty rating	3.92	0.72	312	-0.10^†^	0.02	0.06	–	
(5) Self rating	4.17	0.56	291	0.05	-0.04	0.12^∗^	0.26^∗∗^	–
(6) Opinion	4.45	0.84	319	-0.03	0.36^∗∗^	0.07	-0.08	0.05

^∗^*p* < 0.05; ^∗∗^*p* < 0.01; ^†^*p* < 0.10.

ANOVA was used to test whether the experimental manipulation resulted in significantly different outcome measures (see **Table 3**). Results show that TA differed significantly according to experimental group (*F* = 3.96, *p* < 0.02). Subject TA in the structured behavioral past group (mean = 3.05, SD = 1.28, *n* = 125) and structured future group (mean = 3.07, SD = 1.11, *n* = 94) was significantly higher versus subject TA in the unstructured interview (mean = 2.64, SD = 1.26, *n* = 96). Scheffe’s *post hoc* test indicates that each of the three groups differed significantly from the other groups (*p* < 0.05).

**Table 3:** Study 1 ANOVA of low, moderate, and high ambiguity conditions.

	*F*	(1) Structured past (low) *n* = 125	(2) Structured future (moderate) *n* = 94	(3) Unstructured (high) *n* = 96	Scheffé’s result
(1) TA	3.96^∗^	3.07 (1.11)	3.07 (1.11)	2.64 (1.26)	1, 2 vs. 3^∗^
(2) Self-efficacy	4.11^∗^	2.70 (5.68)	4.42 (4.79)	4.72 (5.49)	1 vs. 3^∗^
(3) Faculty ratings	2.89^†^	3.99 (0.63)	3.77 (0.82)	3.96 (0.73)	2 vs. 1, 3^†^
(4) Self-ratings	0.37	4.14 (0.68)	4.17 (0.53)	4.21 (0.47)	n.s.
(5) Opinions	1.68	4.42 (1.25)	4.55 (0.51)	4.35 (0.66)	n.s.

^∗^*p* < 0.05; ^†^*p* < 0.10.

Self-efficacy change varied according to experimental group (*F* = 4.11, *p* < 0.02), with unstructured interview participants reporting the lowest change in self-efficacy (mean = 2.70, SD = 5.68, *n* = 90). Subjects in the structured future condition reported a higher self-efficacy increase (mean = 4.42, SD = 4.79, *n* = 95), and those in the structured past condition reported the highest self-efficacy increase (mean = 4.72, SD = 5.49, *n* = 127). A Scheffe’s *post hoc* test revealed that the difference between the structured past condition and the unstructured condition was significant (*p* < 0.02).

Faculty ratings varied at a marginal significance level according to experimental group (*F* = 2.89, *p* < 0.06). Faculty ratings were lower for the structured future condition (mean = 3.77, SD = 0.82, *n* = 92) vs. the unstructured condition (mean = 3.96, SD = 0.73, *n* = 92) and structured past condition (mean = 3.99, SD = 0.63, *n* = 128). Scheffe’s *post hoc* test indicated that these latter conditions were marginally higher than the structured future condition (*p* < 0.07). Self-ratings did not vary according to experimental group (*F* = 0.37, *p* < 0.69). The overall subject self-ratings were above average (mean = 4.17, SD = 0.56, *n* = 291). Post-assessment opinions also did not differ according to experimental group (*F* = 1.68, *p* < 0.89), with an overall above average rating (mean = 4.45, SD = 0.84, *n* = 319).

Study 1 results suggest that varied situational ambiguity may result in varied TA, self-efficacy increases, and faculty ratings. Low and moderate ambiguity conditions experienced the most positive changes vs. the high ambiguity condition.

## Study 2

The purpose of the second study was to assess the TA of a sufficiently similar student sample, but without the influence of the assessment and its manipulations. The students in this sample were also in the capstone management class, but completed the TA questionnaire without assessment participation. Therefore, these students did not perceive themselves to be a control group or interact with other students who had participated in the assessment (*n* = 103). We expected that Study 2 students would have lower TA because they had not participated in the assessment experience that could serve to strengthen tolerance levels.

### Sample

The students completed the TA and self-efficacy questionnaires at approximately the same part of the semester as those in Study 1 did, but during a segment focused on management self-development. The Study 2 and Study 1 samples did not significantly differ in age (*t* = -0.13, *p* < 0.90), work experience (*t* = 1.55, *p* < 0.12), or pre-assessment self-efficacy (*t* = -1.08, *p* < 0.28). The percentage of male participants in Study 1 was 55.7% (*n* = 180), while the percentage of males in Study 2 was 49.0% (*n* = 49). The percentages were not significantly different (χ^2^ = 1.39, *p* < 0.24). The students’ major field of study also did not differ between Study 1 and Study 2 groups (χ^2^ = 0.6, *p* < 0.97). The majority of students were management majors in Study 1 (*n* = 213, 68.7%) and Study 2 (*n* = 68, 69.4%). A large portion were also management minors in both Study 1 (*n* = 56, 18.1%) and Study 2 (*n* = 18, 18.4%). Remaining students majored in other business fields in both Study 1 (*n* = 41, 13.2%) and Study 2 (*n* = 12, 12.2%).

### Methods and Measures

Reliabilities were again acceptable for the same Study 1 measures of self-efficacy (only the pre-assessment measure from Study 1, 0.91) and TA (0.84). As in Study 1, PCA resulted in a one-factor solution for TA (eigenvalue = 3.05, 60.97% of variance).

### Results

The Study 2 sample TA differed significantly from the Study 1 TA (*t* = 3.02, *p* < 0.001). Study 1 participants’ TA levels, with all experimental manipulations combined (mean = 2.93, SD = 1.24, *n* = 315), were significantly higher than Study 2 participants’ TA levels (mean = 2.49, SD = 1.37, *n* = 98). Self-efficacy did not significantly differ between the two groups (*t* = -1.08, *p* < 0.28), with similar mean values for Study 1 (mean = 82.31, SD = 9.49, *n* = 319) and Study 2 (mean = 83.51, SD = 10.32, *n* = 101). TA and self-efficacy were not correlated (*r* = 0.11, *p* < 0.26). In Study 1, self-efficacy change was also not correlated with TA, although both constructs appeared to be similarly affected by the experimental manipulations.

**FIGURE 1:**
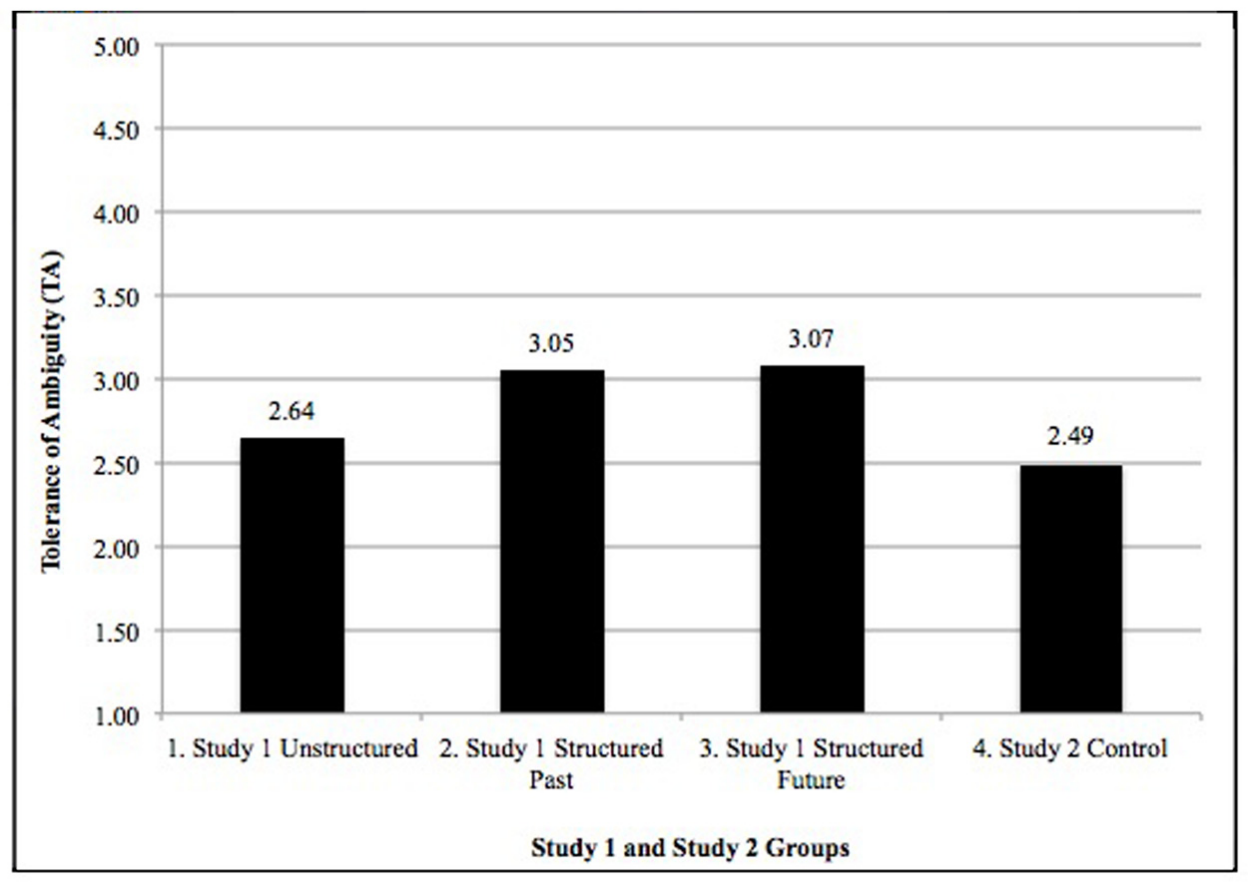
**Tolerance of ambiguity (TA) means compared across Study 1 and Study 2 groups**.

We also analyzed whether TA differed according to four groups consisting of the three Study 1 experimental groups (1–3) and the Study 2 control group (4). TA differed significantly across the four groups (*F* = 5.56, *p* < 0.001). According to Scheffe’s *post hoc* test, the Study 2 control group’s TA was significantly lower than the two structured interview conditions in Study 1, but not significantly lower than the unstructured interview condition in Study 1. **Figure 1** shows the group differences.

Study 2 sought to provide a matched sample for Study 1 that would serve as a control group. This Study 2 group was similar on major demographics as well as being in the same course, having the same majors, and the same level of study as those in Study 1. The Study 2 students, however, were not introduced to the assessment participation and were completing a TA scale as a part of the coursework. Study 2 TA was significantly higher than the combined Study 1 TA. Those who participated in the assessment had an overall lower tolerance. When comparing all groups in the two studies, however, the Study 2 control group and Study 1 unstructured interview (highest ambiguity) reported lowest TA levels. As in Study 1, the structured interview manipulations (both past and future styles of interview) reported highest TA levels. These findings suggest that TA may be malleable and can be increased by exposure to structure and more information in an ambiguous situation.

## Conclusion

Our goal was to use an experimental manipulation in order to contribute to the question of whether TA is a stable trait or a malleable one. The experiment involved senior business students in a capstone class participating in a management skills assessment with a faculty group interview and case analysis. The results here are preliminary, but suggest that TA may be malleable. We conducted two studies, one in which ambiguity was manipulated using structured and unstructured interviews. Less ambiguity appears to have led to higher TA than high ambiguity in a situation of less structure and information.

A limitation of the first study was that there was no information about the pre-experiment TA due to fear of priming the subjects with pre- and post-measures so close together. For additional information, we conducted a second study in which we surveyed a demographically similar group of subjects without the experimental manipulation. These subjects reported TA at the levels of those in Study 1’s high ambiguity (unstructured interview) group, which levels were significantly lower than those in the Study 1 low and moderate ambiguity TA (structured past and future interview). We concluded, therefore, that individual TA may be increased in an ambiguous situation by imposing structure and providing more information.

Subjects who were given more structure and information in the Study 1 assessment also appeared to receive other benefits. Those in the low and moderate ambiguity conditions reported a significantly higher gain in self-efficacy to use management skills, and were also rated more positively by faculty, although at a marginal significance level. Subjects did not vary according to experimental condition in their opinions of the assessment or in their self-ratings, however. The five-point Likert scale for judging performance may have limited the range of self- and faculty ratings, or the view of one’s own performance simply may not be related to the situation’s ambiguity. Individuals may simply judge themselves as on an even ground with others in the assessment, and not relate this to their ability to deal with ambiguity.

Faculty, however, did judge the students differently (marginally), suggesting that either the lessened ambiguity in the structured conditions may have led to higher performance in the interview and case analysis, or the faculty may have been influenced by the experimental manipulation. Those in the structured interview groups may have been influenced by the specific, behavioral answers they received and that created a halo effect over the individual ratings as well. Our inter-rater reliability of faculty and graduate student ratings gives more validity to the single-rater performance measure.

## Implications

Future researchers could measure TA earlier rather than directly before a manipulation or a naturally occurring event and reduce the priming effect, but also improve knowledge about the pre- vs. post-TA levels. There may have been aspects of personality or cognition that we did not measure and that influenced TA. Future researchers could study various subject types. Our sample of BBA senior students may limit the range of some findings, although the students did qualify as adults with work experience. The lack of gender difference is different from what has been found in past studies, however, and this may be due to their fields of study.

Future studies could investigate TA changes over time in the same subject group and include other related personality constructs. This experience may have lasting impact on one’s tolerance in similar situations or create a mindset that is concrete in structured conditions, vs. more malleable in unstructured conditions, for example. Therefore, the short-term versus long-term properties of TA may be important and would be an interesting topic in future research studies.

If TA is malleable and can be increased in otherwise ambiguous situations, training could be designed for dealing with unexpected or unknown decisions at work. Individuals may be able to learn strategies for increasing situational TA that are highly specific to their situations. They also may be able to sustain these increases over time while sharpening their abilities. Most certainly, familiarity with a task or environment would decrease ambiguity, but our findings suggest that a changing or unpredictable environment could result in lower self-efficacy, lower external evaluations, and lower tolerance.

In short, we sought to add knowledge to the question of whether TA is a malleable within the individual. Our results suggest TA may change within the person when faced with varied situations and we encourage further study.

## Conflict of Interest Statement

The authors declare that the research was conducted in the absence of any commercial or financial relationships that could be construed as a potential conflict of interest.

## Footnotes

1 Approval secured from University Human Subjects Committee.
